# Supplementary material for: Fully automatic trace gas plume detection
Source: Sci Rep. 2026 Oct 6;16:35485. doi: 10.1038/s41598-026-70499-1 (PMC13719490; doi:10.1038/s41598-026-70499-1)
Supplement: Supplementary file 1 [file 41598_2026_70499_MOESM1_ESM.pdf]

Supplementary Materials for Fully Automatic Trace Gas Plume Detection

Vít Růžička*, David R. Thompson, Jay E. Fahlen, Amanda M. Lopez, Steven Lu, Chuchu Xiang, Holly Bender, Daniel Jensen, Phil Brodrick, Jake Lee, Brian Bue, Daniel H. Cusworth, Luis Guanter, Adam Chlus, Andrew Thorpe, Robert O. Green
*Corresponding author. Email: ruzicka@jpl.nasa.gov

Text S1. Adapting spectral fitting to other trace gases

We extend the plume transmittance methods of[20] for trace gases other than methane, selecting different spectral ranges according to the absorption features of each target. Figure 3 details this modification. We first calculate column-wise matched filter (CMF) matched filter products using the target signatures of these other trace gas species (Figure 1).

Second, we apply the machine learning models described in Section 2.2 to detect of plume-like shapes in these matched filter products. The models are applied in zero-shot fashion without any adaptation, trained on a dataset of methane sources with just the CH_4-CMF on its input. Since all gases function as passive tracers in the atmosphere, we hypothesize that the plume shapes will in principle be the same for both. In practice, of course, aspects of the CMF product might differ slightly for the different spectral signatures, including variable influence of surface albedo and background clutter.

Third, as with methane, we score each predicted plume with spectral fit metrics adapted to each specific trace gas. The overview of the hyperparameters used for these gases is listed in Table S1. Parameters not listed in this table are kept the same default value for all species. We compare the plume transmittance spectrum to the known target gas absorptions. In each interval, we fit a spectral model based on a polynomial continuum perturbed by some amount of Beer-Lambert absorption by the target species. The polynomial represents any residual difference in surface reflectance that was not removed by the background ratio. The Beer-Lambert absorption gives an estimate of the optical depth. For methane, which has lines that overlap water absorptions in the shortwave infrared, we calculate the effective absorption coefficients at high resolution with an atmosphere and observing geometry appropriate for the scene. For the other target gases, we use absorption coefficients from the MODTRAN line list. We fit a combined model for the plume transmittance including all polynomial coefficients with the plume optical depth.

Table S1. Parameters for plume vetting. Note that in case of NH_3 we obtain three D_{norm} scores according to the used in-band range. MF threshold is used to select low enhancement pixels during background sampling.

	In-band ranges (nm)	Out-band ranges (nm)	MF thr.
CH_4	<2100; 2440>	<381; 1633>, <1692; 2094>, <2441; 2493>	30
NH_3	1: <1498; 1603><br>2: <1952; 2130><br>3: <2123; 2326>	<381; 1498>, <1603; 1922>, <2441; 2493>	300
NO_2	<381; 753>	<753; 1633>, <1692; 2094>, <2441; 2493>	1200
CO	<2278; 2441>	<381; 1633>, <1692; 2094>, <2441; 2493>	300

Text S2. Example NH_3, NO_2 and CO events

Selected examples for NH_3 are on Figure S1, NO_2 on Figure S2 and finally for CO events on Figure S3. Top rows shows the location of the found events on high resolution satellite imagery provided through Google Earth. On the bottom row, we show the computed enhancement product with the vector predicted by our machine learning models. Names of the used EMIT scenes can be uniquely identified by the provided capture time. Particularly striking are the detections of gas plumes over populated areas (2nd and 3rd example in NH_3 and last example in NO_2).

Text S3. Exploring confounder events for NH_3, NO_2 and CO

In Figure S4 we show examples of new types of confounders found for NH_3, NO_2 and CO. We highlight that the spectral fit metrics introduced in this paper are effective for disambiguation between confounder and true point source events.

We believe these examples can be useful to guide future research directions - for example many of NO_2 confounders occur over water bodies, river deltas and oxbows. This could be of potential biological origins, for example as an indication of the chlorophyll *a* (which has a strong absorption feature around 440 nm) and algae or phytoplankton[41]. Computing water masks and filtering out detections over water bodies could be one easy way of addressing these confounders. Certain mineral species also trigger matched filter products for NH_3 and NO_2 (as is also the case of CH_4). In case of CO confounders, lime stone mines (shown on first two scenes) may be triggering the MF enhancement, likely due to the absorption feature of calcium carbonate ($CaCO_3$) around 2300 nm[42].

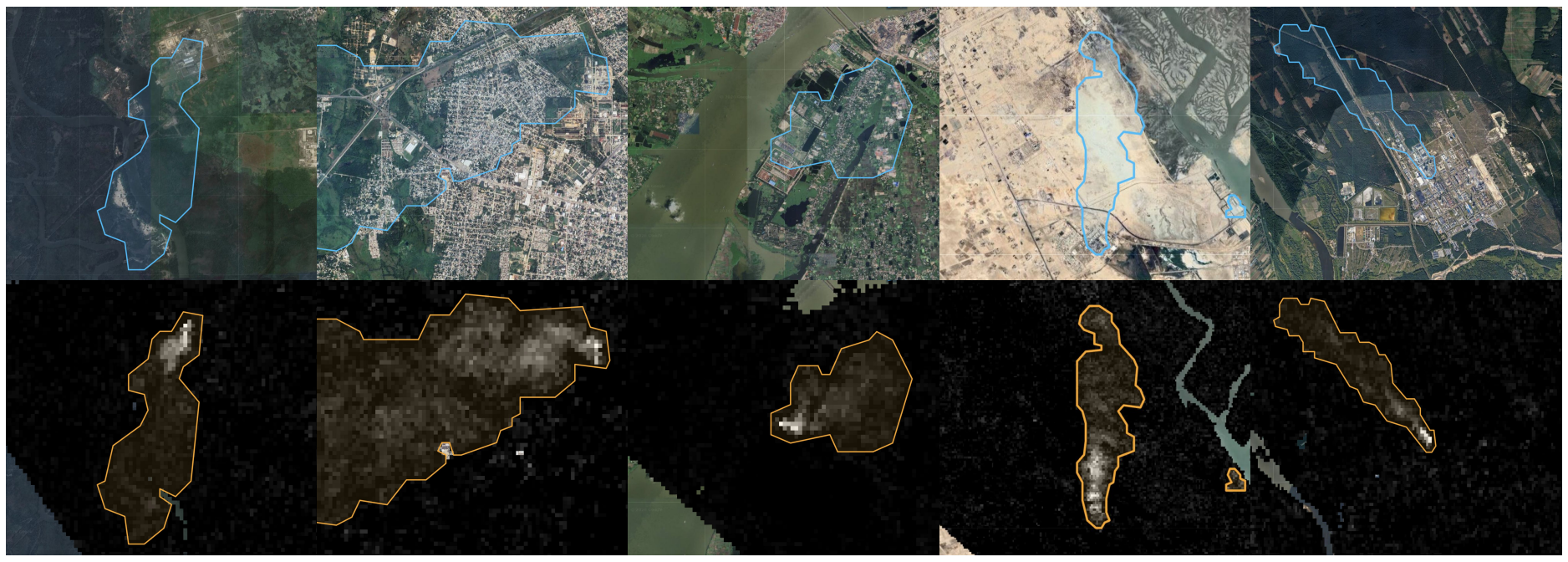

Figure S1. Example NH_3 point sources detected around the world in EMIT data. We highlight the 2nd and 3rd examples, which occur in densely populated regions. From left to right, emission sources include a fertilizer plant in Nigeria, ammonia plant in Mexico, fertilizer plant in Bangladesh, petrochemical plant in Iraq, and urea plant in Poland.

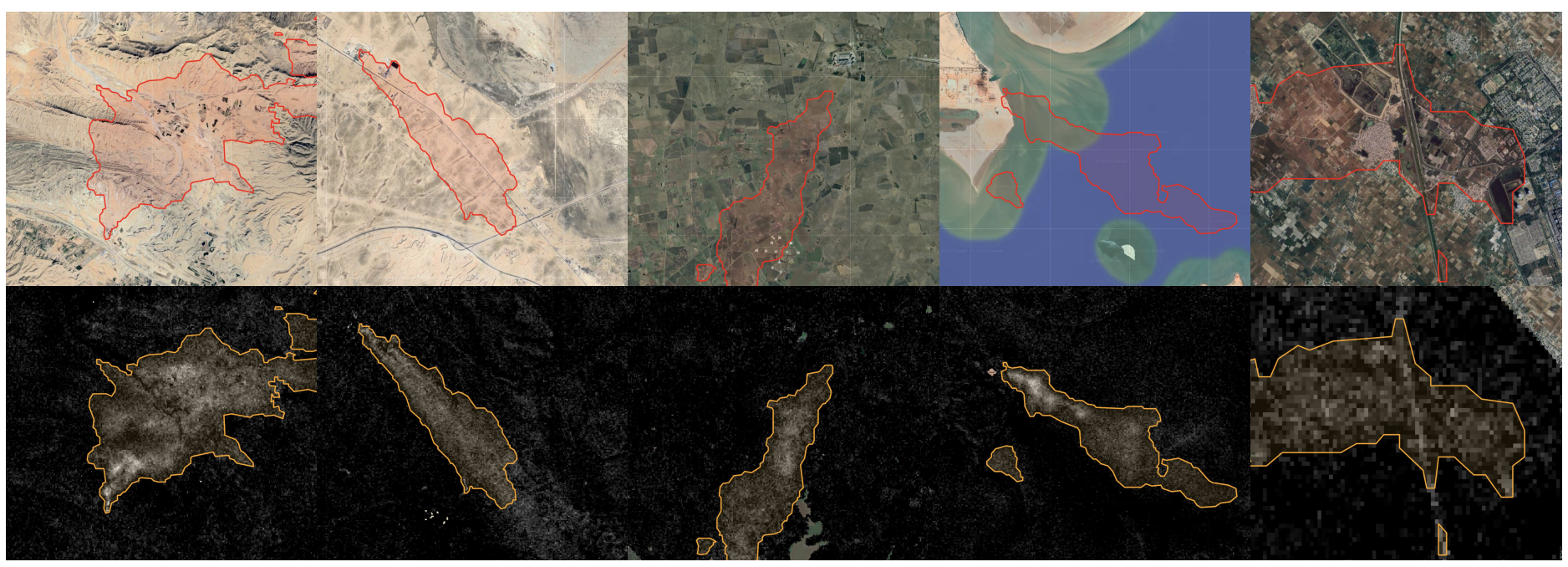

Figure S2. Example NO_2 point sources detected around the world in EMIT data. All emission sources are from power plants.

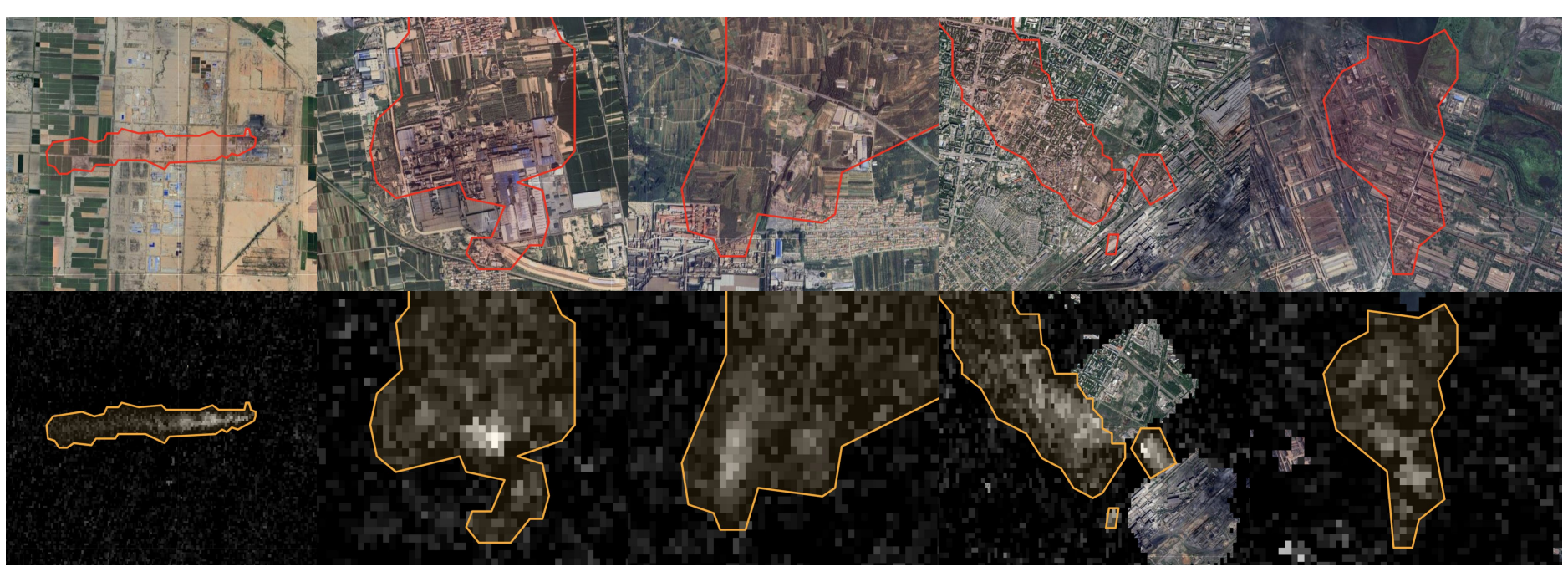

Figure S3. Example CO point sources detected around the world in EMIT data. From left to right, emission sources include two chemical plants in China, possible cement production in China, and steel plants in Kazakhstan and India.

Supplementary Materials References

41. Lee, Z. & Carder, K. L. Absorption spectrum of phytoplankton pigments derived from hyperspectral remote-sensing reflectance. Remote. sensing environment 89, 361–368 (2004).

42. Cao, W. et al. Semi-quantification of the calcium carbonate in marine sediments by visible and near-infrared diffuse reflectance spectroscopy. Geochem. Geophys. Geosystems 25, e2023GC011370 (2024).

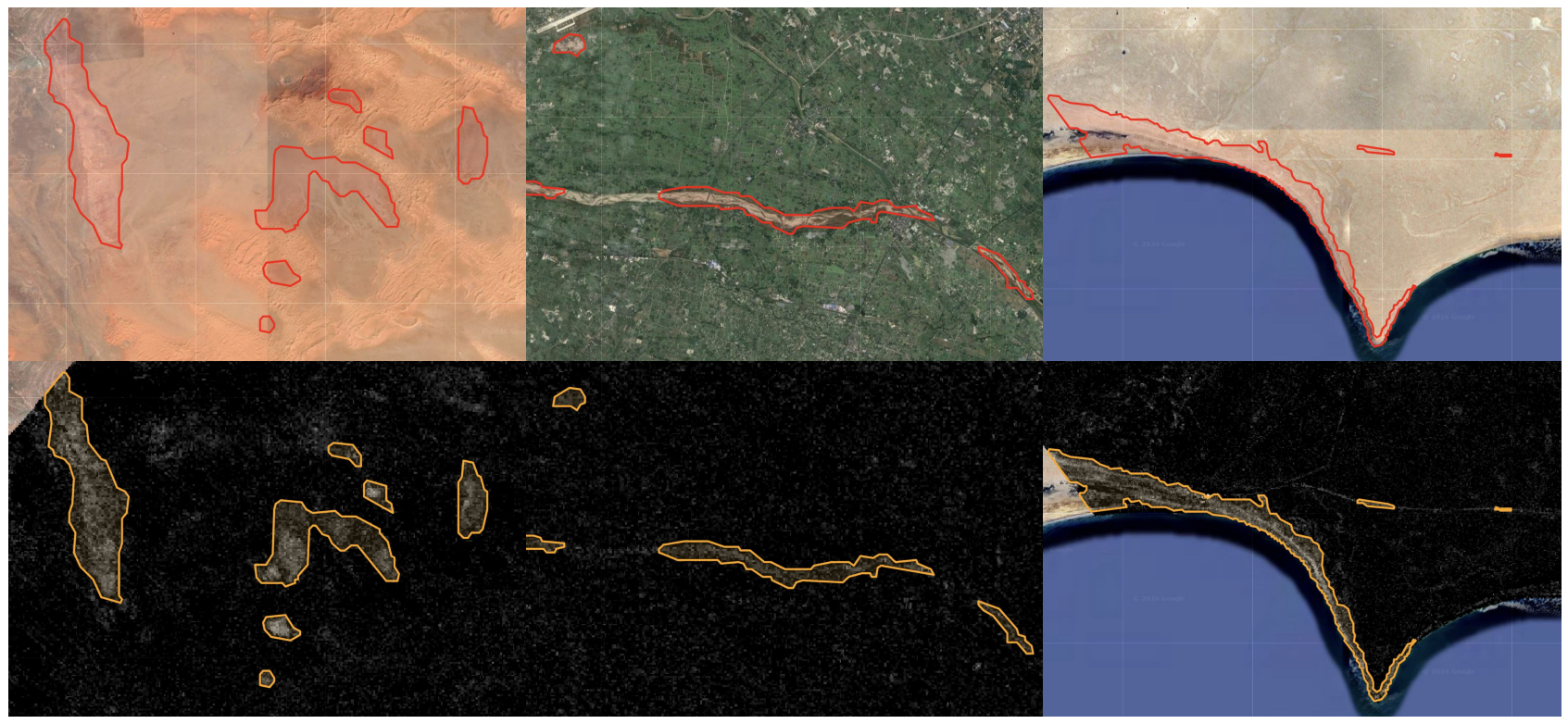

Libya (26.6595, 11.1245),
EMIT 20240204T090348

China (31.0663, 104.0321),
EMIT 20251130T035642

Kazachstan (42.8596, 52.2156),
EMIT 20251003T073526

(a) Example NH_3 confounders.

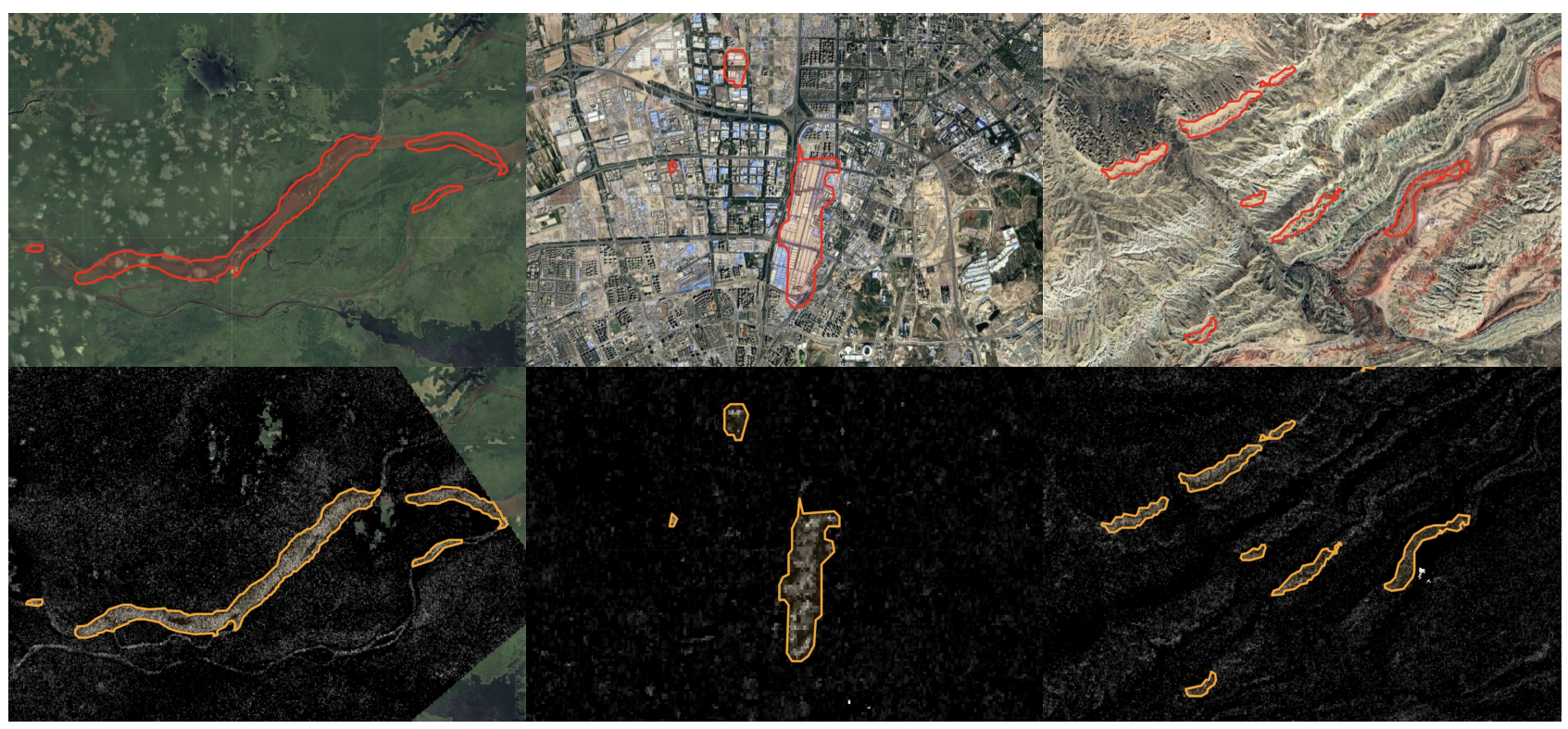

Gabon (-1.0652, 9.3545),
EMIT 20250212T153054

China (43.9218, 87.6128),
EMIT 20240413T075328

Uzbekistan (38.1240, 66.3011),
EMIT 20220901T034318

(b) Example NO_2 confounders.

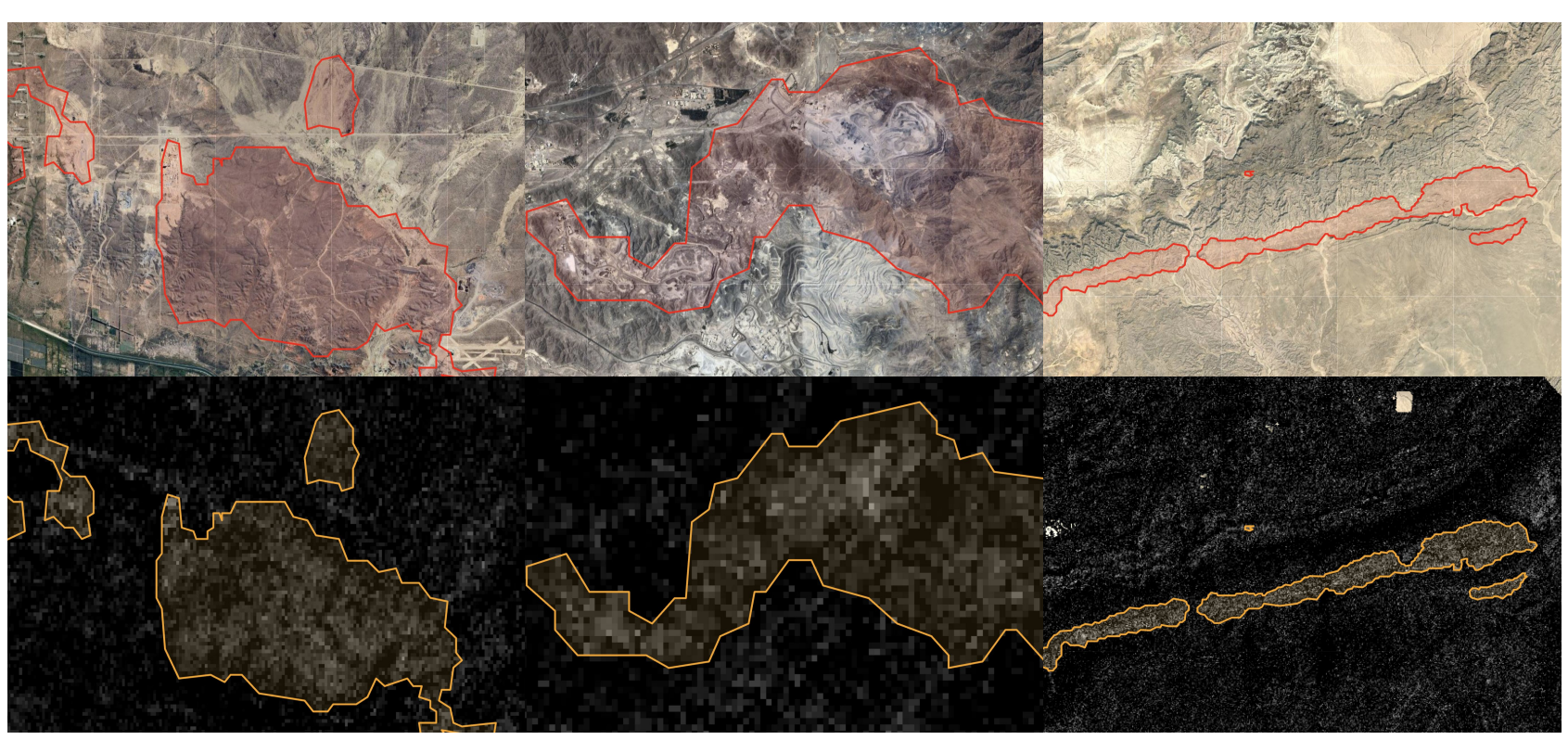

Argentina (-38.9284, -67.8601),
EMIT 20240223T194227

UAE (25.2597, 56.1178),
EMIT 20240621T082746

Pakistan (28.8819, 69.5654),
EMIT 20240418T084009

(c) Example CO confounders.

Figure S4. Example NH_3, NO_2 and CO confounders. While in the case of NH_3 and CO most of these are primarily mineralogical, in the case of NO_2 we observe potential biological triggers. Names of the used EMIT scenes can be uniquely identified by the provided capture time.
